# Copper chelation and interleukin-6 proinflammatory cytokine effects on expression of different proteins involved in iron metabolism in HepG2 cell line

**DOI:** 10.1186/s12858-017-0076-2

**Published:** 2017-01-24

**Authors:** Luca Marco Di Bella, Roberto Alampi, Flavia Biundo, Giovanni Toscano, Maria Rosa Felice

**Affiliations:** 1 Department of Chemical, Biological, Pharmaceutical, and Environmental Sciences, University of Messina, Viale F. Stagno D’Alcontres, 31, 98166 Messina, Italy; 2 Inter University National Group of Marine Sciences (CoNISMa), Piazzale Flaminio, 9, 00196 Rome, Italy

**Keywords:** Iron metabolism, Copper deficiency, Inflammation, Ceruloplasmin

## Abstract

**Background:**

In vertebrates, there is an intimate relationship between copper and iron homeostasis. Copper deficiency, which leads to a defect in ceruloplasmin enzymatic activity, has a strong effect on iron homeostasis resulting in cellular iron retention. Much is known about the mechanisms underlying cellular iron retention under “normal” conditions, however, less is known about the effect of copper deficiency during inflammation.

**Results:**

We show that copper deficiency and the inflammatory cytokine interleukin-6 have different effects on the expression of proteins involved in iron and copper metabolism such as the soluble and glycosylphosphtidylinositol anchored forms of ceruloplasmin, hepcidin, ferroportin1, transferrin receptor1, divalent metal transporter1 and H-ferritin subunit. We demonstrate, using the human HepG2 cell line, that in addition to ceruloplasmin isoforms, copper deficiency affects other proteins, some posttranslationally and some at the transcriptional level. The addition of interleukin-6, moreover, has different effects on expression of ferroportin1 and ceruloplasmin, in which ferroportin1 is decreased while ceruloplasmin is increased. These effects are stronger when a copper chelating agent and IL-6 are used simultaneously.

**Conclusions:**

These results suggest that copper chelation has effects not only on ceruloplasmin but also on other proteins involved in iron metabolism, sometimes at the mRNA level and, in inflammatory conditions, the functions of ferroportin and ceruloplasmin may be independent.

## Background

Iron and copper are cofactors for numerous enzymes and are essential elements for all eukaryotes. They are, however, potentially dangerous because they can react with molecular oxygen generating reactive oxygen species that will damage DNA, lipids and proteins [1–3], and because they are both essential and dangerous their levels are strictly regulated. The copper-containing protein ceruloplasmin has an essential role in iron homeostasis. Its catalytic site has six copper atoms, four of which are involved in iron oxidation [4–6], converting Fe^2+^ to Fe^3+^ without generating reactive oxygen species. In vertebrates, two forms of ceruloplasmin are expressed; the first is mainly produced by hepatocytes and is secreted into the circulation [7–9]. A second form, which is generated by alternative splicing, contains a glycosylphosphatidylinositol (GPI) moiety instead of the normal carboxyl terminal. The GPI anchors ceruloplasmin in the plasma membrane. GPI-Cp was found first in astrocytes where it represents the principal ferroxidase [10, 11]. GPI-Cp, however, is expressed by other cellular types such as leptomeningeal cells, Sertoli cells, and hepatocytes [12–15]. Another important ferroxidase is hephaestin, a transmembrane protein first detected in the small intestine [16, 17]. It mediates iron export from enterocytes to the bloodstream. Hephaestin and the two different forms of ceruloplasmin are suggested to interact with ferroportin, the only known protein involved in ferrous iron export from the cells [18–22]. Fe^3+^ generated by ferroxidase activity, is loaded onto transferrin (Tf), the major iron-containing protein involved in plasma iron transport and distribution within organisms [23–25]. Diferric Tf binds to transferrin receptor 1 (TfR1) present on the plasma membrane of most cell types and in particular on developing red blood cells [26]. Once bound, the Tf(Fe)_2_-TfR1 complex is internalized into an endosome where iron is released from Tf and is then exported to the cytoplasm by divalent metal transporter 1 protein (DMT1) [27–29]. The importance of ceruloplasmin in iron metabolism is demonstrated by the fact that decreases in active ceruloplasmin, as seen in Wilson or Menkes disease, is characterized by a strong accumulation of iron in liver, spleen, and brain [30–34]. Moreover, different studies have highlighted the importance of ceruloplasmin and iron metabolism in pathologies like Alzheimer and Parkinson diseases [35–38].

Systemic iron homeostasis is regulated by different stimuli and, in particular, inflammation can affect the concentration and accumulation of iron in the serum and in different organs [39]. Hepatocytes play a critical role in cellular iron as they are the major storage site for excess iron and are a central regulator of proteins (transferrin, ceruloplasmin and hepcidin) that play an important role in iron homeostasis. In particular, hepatocytes are the principal producers of the secreted peptide hormone, hepcidin. Hepcidin, by binding to the iron exporter ferroportin (Fpn1), induces its degradation resulting in reduced iron uptake from the diet and iron efflux from macrophages [40–42]. Hepcidin mRNA expression is increased by inflammatory cytokines [43, 44]. In particular, IL-6, a proinflammatory cytokine, induces the synthesis of hepcidin and it is responsible of a state of hypoferremia of inflammation [44, 45]. Pro-inflammatory cytokines can also regulate expression of other proteins involved in iron metabolism such as Fpn1, DMT1, TfR1 and ceruloplasmin [46–50].

Although studies have highlighted the effect exerted by copper deprivation or pro-inflammatory cytokines on expression of proteins involved in iron metabolism separately, it is not known if there is a synergistic effect of copper depletion and inflammation. The aim of this study was to analyse the effect of copper chelation and the pro-inflammatory cytokine interleukin-6 (IL-6) on the mRNA and protein levels of different proteins involved in iron metabolism using the human hepatocytoma cell line HepG2 as a model system.

## Methods

### Cell culture and treatment

The hepatocytoma cell line HepG2, kindly provided by prof. M.T. Sciortino (Department of Chemical, Biological, Pharmaceutical and Environmental Sciences, University of Messina, Italy), was grown in Eagle’s minimum essential medium (EMEM) (Lonza) supplemented with 10% Fetal Bovine Serum (Lonza), 1× non-essential amino acids (Lonza), 2 mM L-glutamine (Lonza), 100 μg/ml Streptomycin (Sigma), 100 U/ml Penicillin (Sigma), at 37 °C, and 5% CO_2_. 4×10^5^ cells/ml were seeded in 6 well plates and incubated for 24 h in supplemented medium. Before treatment, cells were washed with PBS and incubated for an additional 16 h in serum-free, antibiotic-free medium, in the presence of 40 ng/ml of IL-6 (Cell Signaling Technology) [51] and/or 300 μM Bathophenanthroline disulfonate (BCS) (Sigma).

### RT-PCR analysis

Total RNA was extracted by EuroGold TriFast reagent (Euroclone) following the manufacturer’s instructions. The concentration and purity of RNA was assayed at 260 nm and 280 nm by a DU 60 Beckman spectrophotometer. One μg of total RNA was retro-transcribed using oligo-dT (EuroClone) and PrimeScript MMLV-RT (Takara, Clontech) at 42 °C for 60 min followed by a denaturation step of 15 min at 70 °C. The primers used for PCR are listed in Table 1. The PCR reactions were run for 30 cycles in MyCycler instruments (BioRad) using EmeraldAmp Hot start DNA polymerase (Takara, Clontech). The PCR conditions adopted were: 98 °C for 10 s, 57 °C for 1 min, 72 °C for 30 s. The PCR amplicons were analyzed by 2.4% agarose gel electrophoresis, and images were acquired by KdS1D system (Kodak) and analyzed by ImageJ 1.47v software (http://imagej.nih.gov/ij). All the intensity values obtained for genes of interest were normalized with respect to β-actin.

**Table 1:** list of primers used in this study

primer	Sequence 5′ → 3′	Reference
Fpn1AB Reverse	CATCCTCTCTGGCGGTTGTG	This study
Fpn1A Forward	TCCATAAGGCTTTGCCTTTCC	This study
Fpn1B Forward	GCATCTGGTTGGAGTTTCAAT	This study
GPI-Cp Reverse	GATTGGGTAGATCACATTCC	[90]
sCp Reverse	CCAATTTATTTCATTCAGCC	[90]
CP Forward	GTCTTTGACCTTATCCCTGG	This study
HAMP Forward	ATGGCACTGAGCTCCCAGAT	This study
HAMP Reverse	TTGCAGCACATCCCACACTTT	This study
β-actin Reverse	CACATCTGCTGGAAGGTGGA	This study
β-actin Forward	CATGAAGTGCGACGTTGACA	This study
	qPCR Primers	
TNF-α Forward	GCAGGTCTACTTTGGGATCATTG	A generous gift of prof. A. Mastino^a^
TNF-α Reverse	GCGTTTGGGAAGGTTGGA	A generous gift of prof. A. Mastino^a^
IL1B Forward	GCGAATGACAGAGGGTTTCTTAG	A generous gift of prof. A. Mastino^a^
IL1B Reverse	CACCTTCAGCTGCCCAGACT	A generous gift of prof. A. Mastino^a^
β-actin Forward	CATTCCAAATATGAGATGCGTTGT	This study
β-actin Reverse	TGTGGACTTGGGAGAGGACT	This study

^a^Department of Chemical Biological Pharmaceutical and Environmental Sciences, University of Messina, Italy

Quantitative Real Time PCR, was performed on the same cDNA using StepOne Plus (Applied Biosystem, LifeTechnologies) and Sybr Premix Ex Taq II (Takara, Clontech) following manufacturer’s instructions and primers listed in Table 1. The amplification was performed at 95 °C for 30 s (1 cycle), 95 °C for 5 s and 60 °C for 60 s (40 cycles). All samples were assayed in duplicate of three independent experiments and the results were normalized to the β-actin housekeeping gene using ΔΔC_T_ method [52, 53].

### Protein extraction and Western blot analysis

To analyze proteins, after specific incubations, cells were washed with PBS and then homogenized in specified lysis buffers. The specific buffer used differed depending on the protein being assayed. For immunodetection of DMT1, H-ferritin subunit, STAT3 and pSTAT3 the lysis buffer was composed of 25 mM MOPS pH 7.4 (Sigma), 150 mM NaCl (Applichem), 1% Triton X-100 (Sigma), and protease inhibitor cocktail (Sigma). The cells were homogenized by passage through a 28 gauge needle several times and left one hour at room temperature, before centrifugation at 15,400 × g for 30 min at 4 °C (Eppendorf 3417R). Total protein concentration of supernatant was assayed by BCA (Pierce) and an equal quantity of total proteins were analyzed by polyacrylamide gel electrophoresis using 16.5% Tris-Tricine SDS-PAGE for DMT1 and FTH1 [54], or 10% SDS-PAGE for STAT3 and pSTAT3, after denaturation at 95 °C for 10 min in the presence of 80 mM Dithiothreitol (DTT). For immunodetection of TfR1 and GPI-Cp cells were homogenized in a buffer composed of 25 mM MOPS pH 7.4 (Sigma), 75 mM NaCl (Applichem), and protease inhibitor cocktail (Sigma) by passage through a 28 gauge needle several times. The homogenate was centrifuged at 15,400 × g for 30 min at 4 °C and the pellet was incubated in extraction buffer composed of 25 mM MOPS pH 7.4, 150 mM NaCl (Sigma), 1% Triton X-100 (Sigma), and protease inhibitor cocktail (Sigma) for one hour before centrifugation as described above. Total protein concentration was assayed by BCA and equal quantities of total protein were separated by 10% Tris-Glycine SDS-PAGE after denaturation at 95 °C for 10 min in the presence of 80 mM DTT. The same membrane protein extraction protocol was used for Fpn1 with the exception that samples were incubated for 30 min at room temperature in the presence of 80 mM DTT before separation on 10% Tris-Glycine SDS-PAGE [55, 56].

After electrophoresis proteins were transferred to FluoroTransW PVDF Membrane (Pall Corporation) by MiniTrans Blot (BioRad) and membranes were blocked with 5% skim milk (Applichem) before incubation overnight with primary antibodies that are listed in Table 2. After washing, the membranes were incubated with HRP-conjugated secondary antibodies and proteins were detected by etaC Westar ECL (Cyagen). The bands were analyzed by ImageJ 1.47v software (http://imagej.nih.gov/ij). The intensity values obtained for proteins of interest were normalized with respect to β-actin protein level.

**Table 2:** list of antibodies used in this study

Target	Dilution	Host	Company
Fpn1	1:1,000	Rabbit	Novus Biologicals
DMT1	1:1,000	Mouse	Novus Biologicals
TfR1	1:5,000	Mouse	Invitrogen
pSTAT3	1:1,000	Rabbit	Cell Signalling
STAT3	1:1,000	Rabbit	Cell Signalling
FTH1	1:1,000	Rabbit	Cell Signalling
Human Ceruloplasmin	1:5,000	Goat	Sigma
β-Actin	1:10,000	Mouse	Sigma
Anti-goat HRP conjugated	1:4,000	Rabbit	Sigma
Anti-mouse HRP conjugated	1:5,000	Goat	Novex, ThermoFisher
Anti-rabbit HRP conjugated	1:4,000	Goat	Novex, ThermoFisher

### Cellular copper and iron concentration

Total cellular homogenates were obtained as described in section 2.3. For copper and iron mineralization, the homogenate was digested with 1:1 volume ratio of 65% HNO_3_ overnight at 60 °C [57].

After a dilution of HNO_3_ to 5%, equal aliquots of samples were used for copper and iron determination by a graphite furnace Perkin Elmer PinAACle 900H atomic absorption spectrophotometer, equipped with the autosampler AS900 and the Lumina cathode lamp (Perkin Elmer). Calibration was against a Cu or a Fe standard curve and the metal content was normalized to total cellular proteins concentration, determined by BCA assay kit, as described in section 2.3.

### Immunodetection and in-gel oxidase activity of secreted Ceruloplasmin

After specific treatments of cells, the medium was collected, concentrated, dialyzed by Centricon YM-50 (Millipore), and proteins were separated by 8% SDS-PAGE in non-denaturing condition for assay of oxidase activity. To assay oxidase activity, gels were incubated in 0.1 M sodium acetate buffer pH 5.0 containing 0.5 μg/ml of o-dianisidine dihydrochloride (Sigma) [58]. Alternatively, samples were incubated under reducing conditions for Western blot analysis and immunodetection (as described above) or gels were stained with Coomassie Blue.

### Statistical analysis

The data were analyzed by GraphPad Prism 5.0. Values are expressed as the mean ± SEM. All assays were performed with samples obtained from six independent experiments. Statistical differences were determined by paired Student’s *t*-test. Differences were considered significant at *p* < 0.05 level.

## Results

### Analysis of *Signal Transducer and Activator of Transcription* 3 (STAT3) transcription factor

**Fig. 1:**
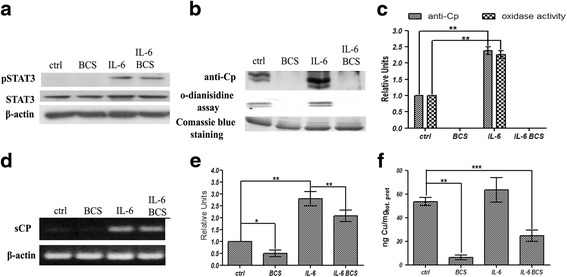
Western blot and RT-PCR analysis. HepG2 cells were treated for 16 h in serum-free medium with 300 μM BCS and/or 40 ng/ml of IL-6. **a** Western blot analysis of pSTAT3, STAT3, and β-actin proteins on total cell extracts as described in methods. **b** Western blot, Coomassie Blue staining of soluble Cp isoform, relative to denaturing SDS-PAGE, and in gel nondenaturing SDS-PAGE enzymatic activity of concentrated and dialyzed culture medium. Equal amounts of total proteins were loaded per lane. **c** relative densitometric analysis. **d** representative image of soluble *Cp* isoform RT-PCR product: after 16 h of treatment, RNA was isolated, reverse transcribed and subjected to PCR. The amplicons relative to soluble *Cp* isoform and *β-actin* were analysed by agarose gel electrophoresis and intensity of bands was determined by ImageJ 1.47v software (http://imagej.nih.gov/ij). The values of intensity relative to soluble Cp were normalized by using the β-actin housekeeping gene, (**e**) densitometric analysis of *Cp* RT-PCR results. **f** graph relative to intracellular copper concentration in HepG2 cells. Cells were extensively washed, lysed as described in methods and used for atomic absorption analysis. The copper content was normalized by cellular total protein concentration. All values are expressed as means ± SEM (*n* = 6). All indicated differences were statistically significant (*p* < 0.05). *p ≤ 0.05; **p ≤ 0.01; ***p ≤ 0.001. Expression levels of control condition were normalized to one, and all values are expressed as relative units

It is known that interleukin-6 is able to induce the phosphorylation and nuclear translocation of the transcription factor STAT3 [59, 60]. The level of pSTAT3 was analysed to verify if the concentration of IL-6 and the period of treatment adopted in the present study were able to evoke a response in the HepG2 cell line. A concentration of 40 ng/ml IL-6 was able to activate STAT3 and the presence of BCS did not affect STAT3 phosphorylation state in control cells or IL-6 treated cells (Fig. 1a).

### Effects of BCS and IL-6 on expression of secreted form of ceruloplasmin and determination of cellular copper concentration

The capacity of BCS to copper deprive cells was investigated by the analysis of the secreted form of ceruloplasmin (Cp), as copper deficiency is known to result in secretion of apoCp that is rapidly turned over [61]. Incubation of HepG2 cells with BCS results in the of loss Cp oxidase activity and immunodetectable Cp (Fig. 1b). IL-6 treatment is able to induce a strong signal of Cp protein with respect to control conditions, yet incubation with BCS results in the disappearance of Cp from the medium. A densitometric analysis comparison highlighted a strong correlation between soluble Cp oxidase activity and immunoreactivity (Fig. 1c). *Cp* mRNA levels were measured to determine if the decrease in Çp protein resulted from a decrease in *Cp* mRNA. Incubation with BCS resulted in a slight but statistically significant decrement of *Cp* mRNA compared to control conditions (Figs. 1d, and e). In contrast, treatment with IL-6 caused a threefold induction of *Cp* mRNA that was only slightly reduced by incubation with BCS, indicating that the absence of Cp in the media of cells treated with BCS was largely due to either a slower rate of protein secretion or degradation of the apo form of secreted Cp and was not the result of downregulation of gene expression.

To exclude a secondary effect exerted by BCS that is independent of copper deficiency, we have determined by atomic absorption copper intracellular concentration and, in accord with the results reported above, the treatment of HepG2 cells with BCS induces a strong decrement of copper content and the cotreatment with IL-6 has only a slight positive effect (Fig. 1f).

### Effects of BCS and IL-6 on expression of GPI-anchored form of ceruloplasmin

**Fig. 2:**
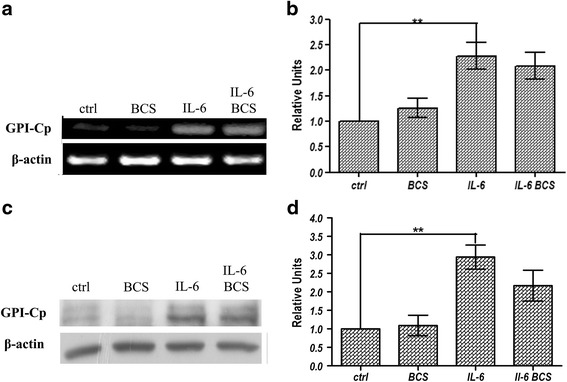
RT-PCR and Western blot analysis of GPI-Cp expression levels. HepG2 cells were treated for 16 h in serum-free medium with 300 μM BCS and/or 40 ng/ml IL-6. **a** and **b** after 16 h of treatment, RNA was isolated, reverse transcribed and subjected to PCR. The amplicons relative to *GPI-Cp* isoform and *β-actin* were analysed by agarose gel electrophoresis and intensity of bands was determined by ImageJ 1.47v software (http://imagej.nih.gov/ij). The values of intensity relative to GPI-Cp were normalized by using the β-actin housekeeping gene. **c** representative image of GPI-Cp isoform protein relative to membrane proteins extracts analysed by western blot. **d** densitometric analysis of GPI-Cp isoform protein. The values are normalized by β-actin protein level. All values are expressed as means ± SEM (*n* = 6). All indicated differences were statistically significant (*p* < 0.05). *p ≤ 0.05; **p ≤ 0.01; ***p ≤ 0.001. Expression levels of control condition were normalized to one, and all values are expressed as relative units

In addition to the secreted form of Cp, hepatocytes express GPI-anchored Cp [15]. The effect of BCS alone or in combination with IL-6 on the GPI-anchored Cp was also investigated. At the transcriptional level (Fig. 2a and b), IL-6 induced a strong induction in *GPI-Cp* mRNA level compared to control cells. Treatment with BCS did not affect transcription in either control cells or in the IL-6 treated cells, indicating a behaviour very similar to that observed for expression of secreted Cp. The presence of BCS did not affect the amount of GPI-Cp present at the plasma membrane (Fig. 2c, and d). Unfortunately, the level of GPI-Cp was too low to assess enzymatic activity.

### Effect of BCS on HAMP and Fpn1 expression

**Fig. 3:**
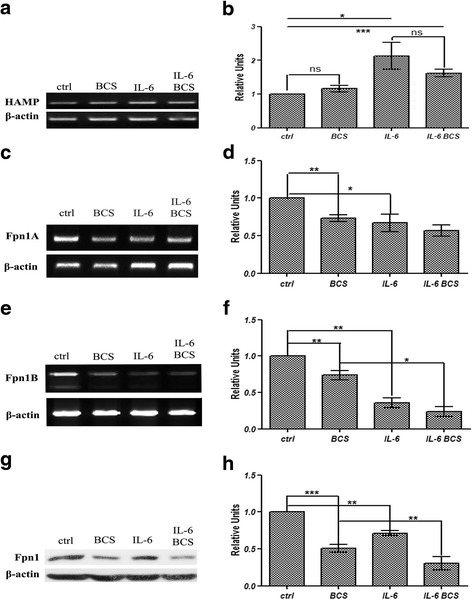
RT-PCR analysis of *HAMP* gene, and RT-PCR and western blot analysis of Fpn1 expression levels. HepG2 cells were treated for 16 h in serum-free medium with 300 μM BCS and/or 40 ng/ml IL-6. **a** after 16 h of treatment, RNA was isolated, reverse transcribed and subjected to PCR. The amplicons relative to *HAMP* and *β-actin* genes were analysed by agarose gel electrophoresis and intensity of bands was determined by ImageJ 1.47v software (http://imagej.nih.gov/ij). The values of intensity relative to HAMP were normalized by using the β-actin housekeeping gene. **b** densitometric analysis of *HAMP* gene RT-PCR results (**c**) representative image of *Fpn1A* isoform RT-PCR product analysed as described above and (**d**) densitometric analysis relative to *Fpn1A* RT-PCR results. The values were normalized by using the β-actin housekeeping gene. **e** representative image of *Fpn1B* isoform RT-PCR product analysed as described above and (**f**) densitometric analysis relative to *Fpn1B* RT-PCR results. The values were normalized by using the β-actin housekeeping gene. **g** representative image of Fpn1 protein immunoblot result relative to membrane proteins extracts. Equal amounts of proteins were loaded per lane. **h** densitometric analysis of Fpn1 protein. The values are normalized to β-actin protein level. All values are expressed as means ± SEM (*n* = 6). All indicated differences were statistically significant (*p* < 0.05). *p ≤ 0.05; **p ≤ 0.01; ***p ≤ 0.001. Expression levels of control condition were normalized to one, and all values are expressed as relative units

Studies have shown a functional relationship between Cp and Fpn1 in which Cp is required to convert Fpn1-exported Fe^2+^ to Fe^3+^ for binding to Tf. Some studies have shown a physical relationship between GPI-Cp and Fpn1 [62, 63]. Based on these results we examined the effects of BCS and IL-6 on *Fpn1* and *HAMP*, the hepcidin gene. IL-6 was able to induce transcription of *HAMP* as previously reported [44], while the presence of BCS did not affect its expression level (Fig. 3a, and b). These results show the effect of BCS is specific for *Cp* expression but not for *HAMP* expression.

At the transcriptional level, expression of the two spliced variant forms of *Fpn1*, *1A* and *1B* [64, 65] (Fig. 3c, d, e, and f) were both decreased by BCS or IL-6. BCS had a similar effect on both isoforms while the negative effect of IL-6 is less evident in variant *1A* (50%) versus variant 1B (30%) (Fig. 3d, and f). Incubation of HepG2 cells with both IL-6 and BCS resulted in a small additive decrease, however, it did not reach statistical significance. A difference in the amount of Fpn1 protein was also observed when cells were treated with BCS or IL-6 (Fig. 3g, and h). Fpn1 levels were decreased 50 or 30% respectively. Further, incubation with BCS and IL-6 resulted in an additional protein decrement, which was statistically significant, indicating an additive effect of the two substrates. These experimental results highlight that the effect of this pro-inflammatory cytokine and copper chelation can negatively regulate Fpn1 expression.

### Effects of BCS and IL-6 on TNF-α and IL-1β expression

To test if the effect exerted by BCS alone or in combination with IL-6 was direct or indirect by production of other pro-inflammatory cytokines, the mRNA level of *TNF-alpha* and *IL-1B* was also assayed by qPCR. Unfortunately, the C_T_ values relative to these two classes of mRNA were very low (C_T_ 36–40) respect to *β-actin* mRNA level, and were not considered for further analysis.

### TfR1, and DMT1 expression

**Fig. 4:**
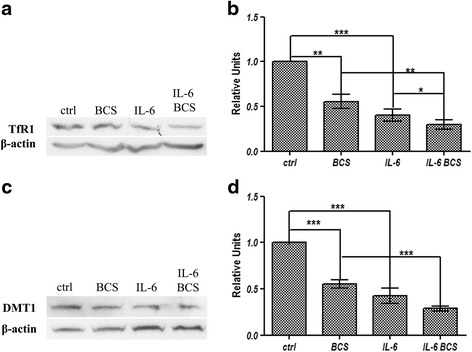
Western blot analysis of TfR1, and DMT1. HepG2 cells were treated for 16 h in serum-free medium with 300 μM BCS and/or 40 ng/ml IL-6. **a** representative image of TfR1 protein immunoblot relative to membrane proteins exstracts. Equal amounts of proteins were loaded per lane. **b** densitometric analysis of TfR1 protein. **c** representative image of DMT1 protein immunoblot relative to total cell extracts, after electrophoresis on 16.5% Tris-Tricine SDS-PAGE (**d**) densitometric analysis of DMT1 protein. The values are normalized to β-actin. All values are expressed as means ± SEM (*n* = 6). All indicated differences were statistically significant (*p* < 0.05). *p ≤ 0.05; **p ≤ 0.01; ***p ≤ 0.001. Expression levels of control condition were normalized to one, and all values are expressed as relative units

Hepatocyte iron uptake through TfR1 and DMT1 is important in conditions of iron deficiency and it is also important under culture conditions in which the amount of iron is limited. For these reasons, the expression levels of these two proteins were investigated under copper chelation and proinflammation. Treatment of HepG2 cells with BCS resulted in a 50% decrease in TfR1 protein levels (Fig. 4a and b) and IL-6 had almost the same effect. Incubation of cells with both BCS and IL-6 led to a further decrease of TfR1 indicating an additive effect. Given the functional relationship of TfR1 and DMT1 in TfR1-mediated iron uptake, the levels of DMT1 were also analysed. Incubation of HepG2 cells with BCS or IL-6 resulted in about a 50% decrement of DMT1 protein, while treatment with both BCS and IL-6 led to an additional decrement, although it did not reach statistical significance (Fig. 4c, and d).

### FTH1 expression and cellular iron concentration

**Fig. 5:**
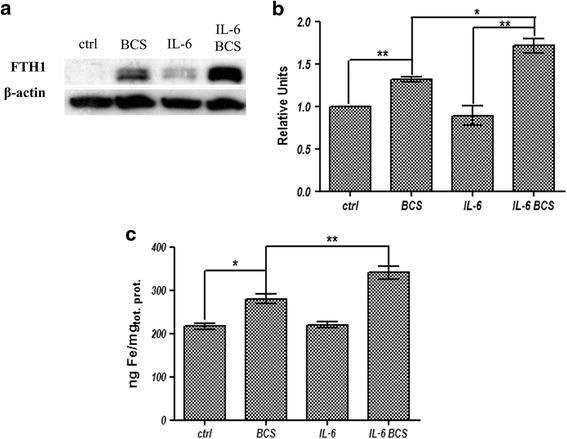
FTH1 expression levels and intracellular iron concentration. **a** representative image of FTH1 protein immunoblot relative to total cell extracts, after electrophoresis on 16.5% Tris-Tricine SDS-PAGE. **b** densitometric analysis of FTH1 protein. The values are normalized to β-actin. **c** graph relative to intracellular iron concentration in HepG2 cells. Cells were extensively washed, lysed as described in methods, and used for atomic absorption analysis. The iron content was normalized by cellular total protein concentration. All values are expressed as means ± SEM (*n* = 6). All indicated differences were statistically significant (*p* < 0.05). *p ≤ 0.05; **p ≤ 0.01; ***p ≤ 0.001. Expression levels of control condition were normalized to one, and all values are expressed as relative units

To test if the experimental conditions affected intracellular iron level, we examined ferritin heavy chain (FTH1) protein levels, an indicator of cytosolic iron. The treatment of cells with IL-6 did not affect FTH1 levels. In contrast, BCS treatment resulted in an increase in FTH1 levels suggesting an increase of cellular iron content (Fig. 5a, and b). Further, the addition of IL-6 together with BCS increased FTH1 protein levels suggesting the intracellular iron levels are greatly increased in copper chelation and proinflammatory conditions. To exclude secondary effects, the cellular iron concentration was determined and, as shown in Fig. 5c, the concentration of iron is coherent with ferritin protein amounts, indicating that BCS is able to induce an increase of intracellular iron concentration.

## Discussion

The copper-containing protein Cp has a key role in iron metabolism and its activity and level relies on appropriate copper acquisition. Accumulation of newly synthesized Cp is dependent on copper availability, as the stability of the apoprotein is severely reduced [61, 66, 67]. In Wilson Diseases caused by a mutation in *ATP7B* gene, a Golgi copper transporter, Cp is produced in the apo-form that is secreted in the blood stream where it is rapidly degraded [68]. Decreased active Cp results in iron accumulation in liver and other organs due to a failure to export cellular iron [30–34]. Our study in the HepG2 cell line confirms that a deficiency of copper induces a strong reduction in the secreted form of Cp. Treatment of cells with IL-6 led to a strong induction in *Cp* mRNA and protein levels, consistent with previously published data [46, 47, 69]. The IL-6 induction of *Cp* mRNA, however, was not able to reverse the negative effect on protein secretion exerted by BCS. Of interest is that our results showed that the presence of BCS had minimal influence on stability of GPI-Cp present on the plasma membrane; unfortunately, we were not able to demonstrate a linear correlation between the amount of protein present and its enzymatic activity. These data are in accord with Mostad et al. [14], who demonstrated that copper deficiency has different effects on GPI-Cp protein level in different organs. Copper deficiency in the spleen induces a strong decrement of GPI-Cp protein levels, while only a slight reduction of the protein was found in liver. The different response of the two Cp isoforms to a copper deprivation state could be explained by different kinetic of secretion or degradation rates of the apoprotein dependent on tissue type.

Our results suggest that copper deficiency has an effect on other proteins involved in iron metabolism. It is known that cellular export of Fe(II) by Fpn1 requires Cp to oxidize Fe^2+^ to Fe^3+^. Studies using transfected C6 and HeLa cells showed that Cp activity is necessary for the stability of plasma membrane Fpn1 [63, 70, 71], and an interaction between the two proteins was also hypothesized [62]. The results reported in this study highlight that Fpn1 is only partially influenced by GPI-Cp protein amount; in fact, in conditions in which cells are treated with BCS, the decrement observed for Fpn1 protein is much more pronounced than that observed for GPI-Cp. This discrepancy can be explained considering the enzymatic activity rather than the protein amount. As mentioned, we do not know if the GPI-Cp protein present on the plasma membrane is also enzymatically active. A slight correlation is seen comparing Fpn1 and sCp protein amounts. The differences observed between our results and the reported published data could be explained with the use of different experimental models. In glioma cell lines the GPI-Cp is the isoform that is mostly highly expressed while in hepatocytes sCp is the most highly expressed isoform [11]. Different experimental models have reported some contrasting results such as animals fed a copper-deficient diet showed an increment of Fpn1 protein when whole liver was analysed. This apparent discrepancy could be due to a different response to the same stimuli between the different cells present in this organ, e.g., Kupffer cells and hepatocytes [14, 72, 73].

To determine if copper deficiency could affect Fpn1 levels by inducing hepcidin we assayed *HAMP* mRNA levels. In our cells, *HAMP* mRNA levels were not affected by copper chelation. In contrast, copper chelation affected *Fpn1* transcripts including both *Fpn1A* and *Fpn1B* mRNA variants. To determine if the decrease observed was linked to a post-transcriptional regulation mechanism mediated by intracellular iron concentration, H-ferritin subunit protein was assayed as a measure of cellular iron content. Our results showed increased levels of H-ferritin suggesting an increase in cytosolic iron concentration. This result was confirmed by the determination of cellular iron concentration. Increased intracellular iron would be expected to increase *Fpn1* translation (IRP) and mRNA stability (mR485-3p), as increased Fpn1 activity is required to export cellular iron [74–76]. The finding that copper chelation leads to increased cellular iron retention and decreased *Fpn1* mRNA suggests a novel mechanism of Fpn1 regulation. The response of HepG2 cell line to BCS is indicative of a state in which the cells protect themselves from the accumulation of intracellular iron, probably because a not functional ceruloplasmin could cause a condition of iron overload. For this reason, it is possible that in the first period of treatment, ferroportin is downregulated causing an increase of cellular iron concentration. As consequence, TfR1-mediated iron uptake is also reduced. Some studies have reported that hepcidin activity can be dependent on copper availability [77]; in fact, it has an “ATCUN” (amino-terminal Cu-Ni)-binding motif in the N-terminal of the mature protein capable to bind copper and nickel, even if a recent study has questioning this possibility [77–79]. Tselepis et al. highlighted that the incapacity of hepcidin to bind copper, drastically reduce the capacity of hepcidin to induce ferroportin degradation [77]. Considering the results reported in this study and the possibility that hepcidin is not able to reduce ferroportin protein amount in condition of copper deficiency, a transcriptional downregulation of ferroportin can contrast a potential iron overload.

The apparent functional relationship between Fpn1 and Cp appears to break down in the face of inflammatory stimuli. *Cp* mRNA isoforms are strongly upregulated by IL-6, while *Fpn1A* and *Fpn1B* mRNAs seem to be downregulated. This effect is also seen on the protein level. The lower level of Fpn1 protein might be explained in part due to the post-translational hepcidin-mediated degradation mechanism [41], as hepcidin is upregulated in inflammation [44]. Our data confirm that in HepG2 cells treatment with IL-6 strongly induces *HAMP* gene expression. However, independent of post-translational regulation, our data show that IL-6 reduces *Fpn1* mRNA. These results are consistent with published data, which demonstrated that IL-6 is able to downregulate *Fpn1* levels in the HepG2 cell line [48] and upregulate the mRNA level of *sCp* [46, 47]. We demonstrate that the GPI-Cp isoform is also upregulated and the protein level of the two isoforms follow the same behaviour. The findings that IL-6 results in increased Cp levels but decreased Fpn1 indicates that the functions of these two proteins are not obligatorily linked together. As mentioned above, it is reported that treatment with IL-6 causes an increase in Cp mRNA level, probably in part by the transcription factor FOXO1 [47]. This protein is involved in cellular response to oxidative stress and upregulation of Cp can enter in the mechanism of correlation between oxidative stress and metal metabolism [80, 81]; in fact, CP ferroxidase activity is important in the loading of Fe(III) on transferrin, reducing the deleterious effect of Fe(II) oxidation and production of radical oxygen species [23, 82]. In this way, Cp enters in the circuit to limit NTBI (non-transferrin bound iron) in the serum with hepcidin that is strongly upregulated in IL-6 induced inflammation and, with its activity, limits the presence of iron in the plasma [43]. In addition to ferroxidase activity, Cp has other functions as Cu(I) oxidation [83], NO-oxidase and NO_2_^−^synthase [84], and superoxide dismutase [85]. Moreover, an interaction between Cp and myeloperoxidase (MPO) was also demonstrated and it is supposed that Cp inhibits prooxidant activity of MPO [86]; in fact, in systemic vasculitis, the interaction between Cp and MPO is prevented by autoantibodies against MPO [87]. In vitro experiments have highlighted an interaction between Cp, MPO and lactoferrin (Lf). This ternary complex has different functions as reduce the activity of MPO, incorporate Fe(III) on Lf and protect Cp from proteolytic cleavage [88]. For these reasons, Cp can have a fundamental role in inflammation conditions and in autoimmunity diseases.

The dysfunction of cellular iron export resulting from copper chelation has an effect on TfR1-mediated iron delivery, resulting in decreased expression of TfR1 and DMT1. These results are consistent with published studies in which copper deficiency led to a decrement in TfR1 protein in the liver [73, 89].

## Conclusions

In summary, here we have demonstrated, using a hepatoma cell line, that IL-6 results in increased Cp levels and decreased Fpn1, indicating that the functions of these two proteins are not obligatorily linked together.

Moreover, we have demonstrated that copper chelation has effects not only on Cp but also on other proteins involved in iron metabolism, sometimes at the mRNA level.
